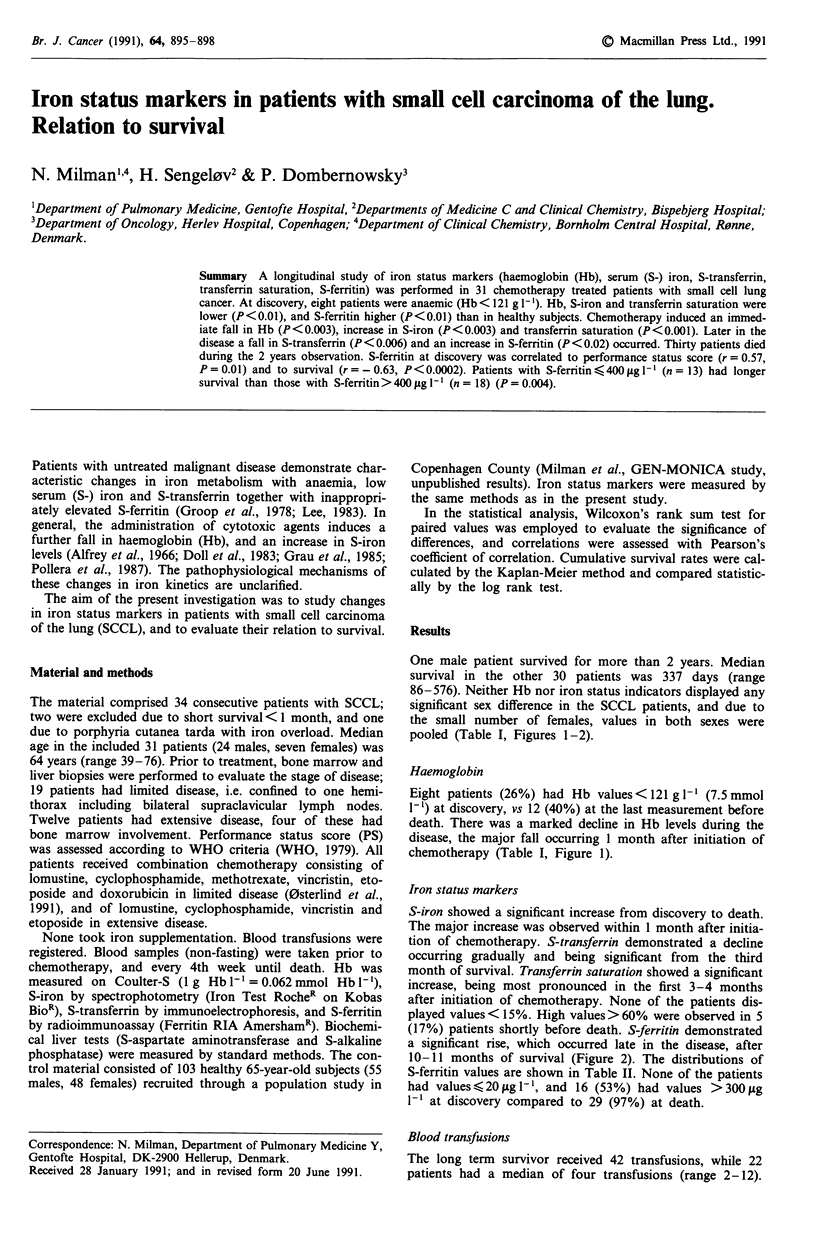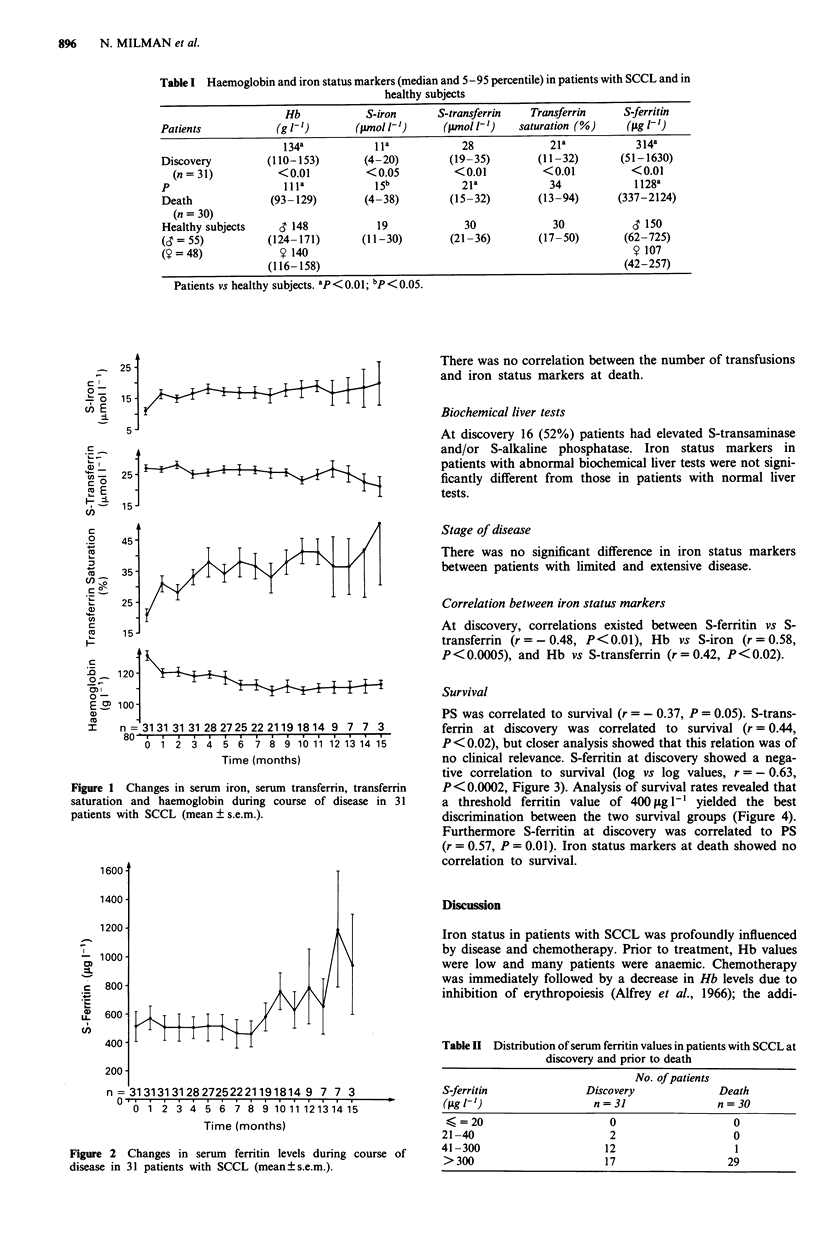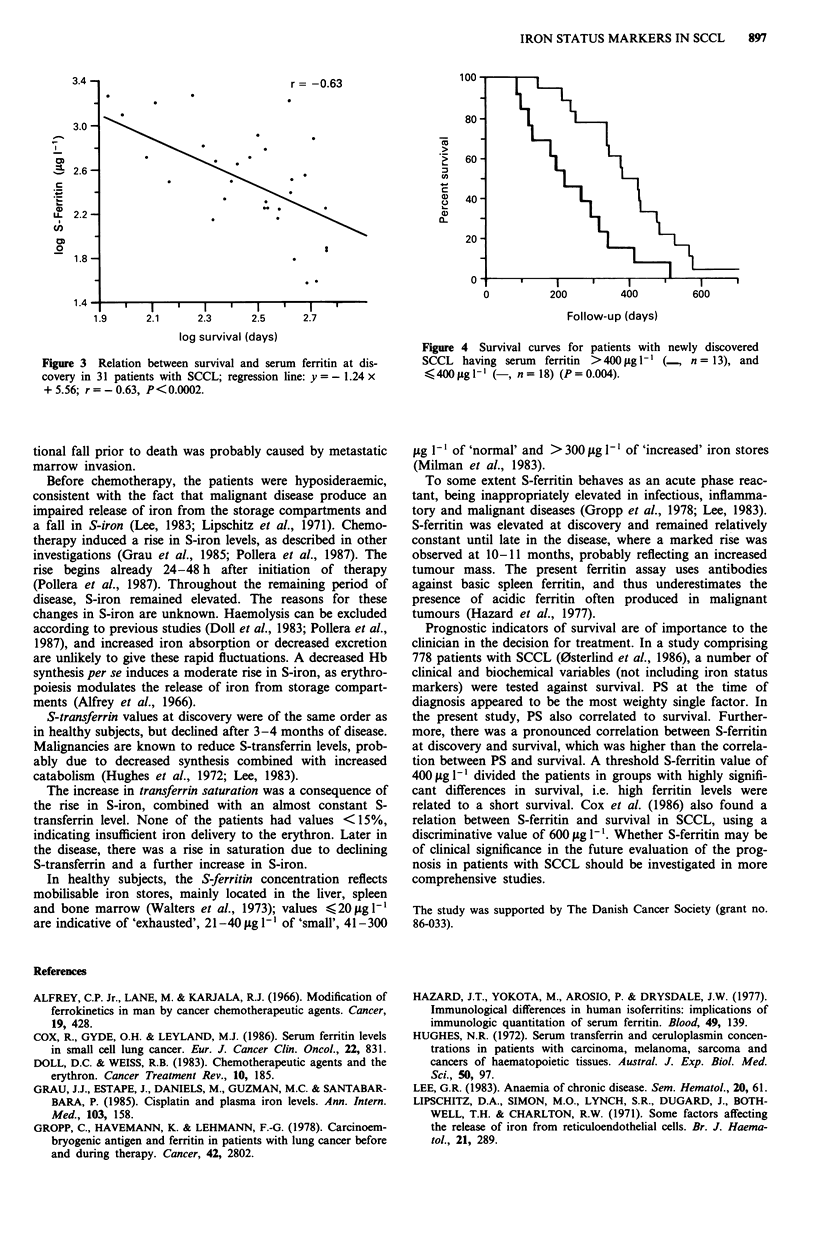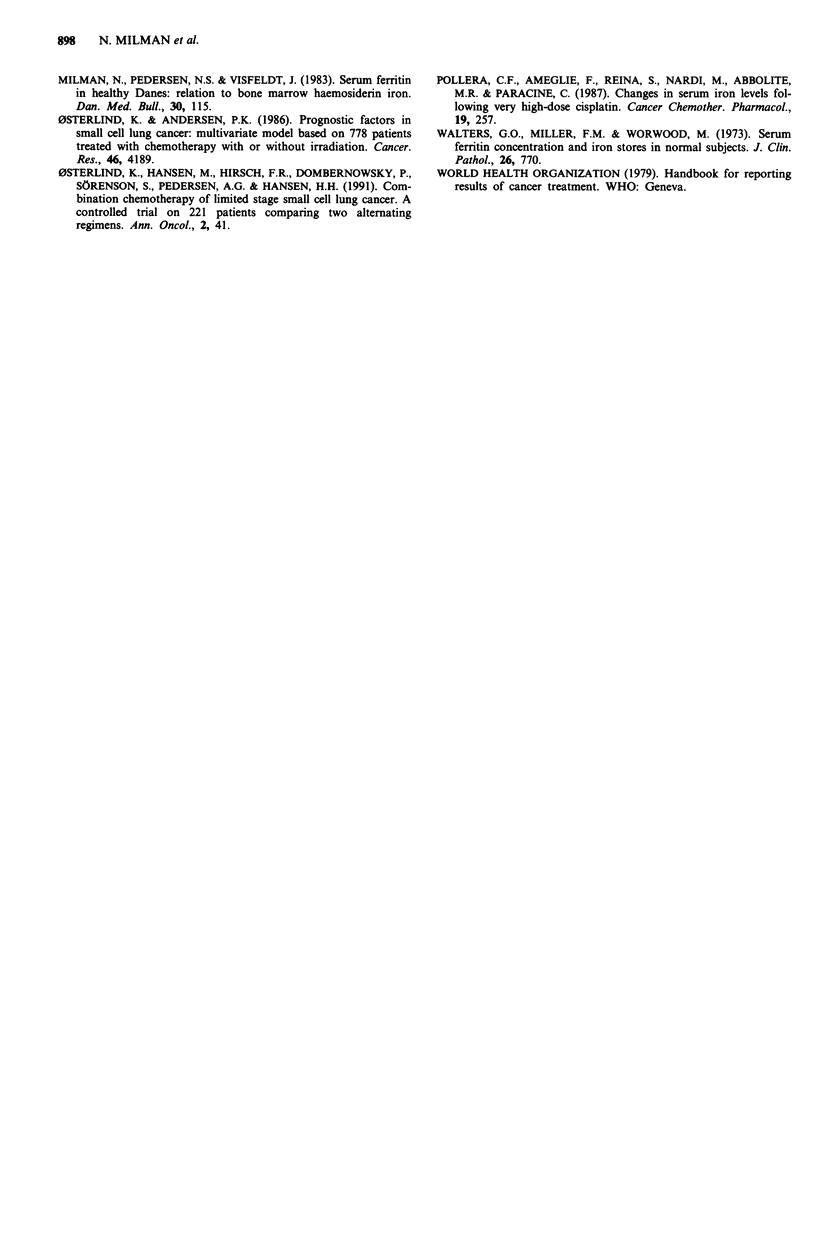# Iron status markers in patients with small cell carcinoma of the lung. Relation to survival.

**DOI:** 10.1038/bjc.1991.421

**Published:** 1991-11

**Authors:** N. Milman, H. Sengeløv, P. Dombernowsky

**Affiliations:** Department of Pulmonary Medicine, Gentofte Hospital, Hellerup, Denmark.

## Abstract

A longitudinal study of iron status markers (haemoglobin (Hb), serum (S-) iron, S-transferrin, transferrin saturation, S-ferritin) was performed in 31 chemotherapy treated patients with small cell lung cancer. At discovery, eight patients were anaemic (Hb less than 121 g l-1). Hb, S-iron and transferrin saturation were lower (P less than 0.01), and S-ferritin higher (P less than 0.01) than in healthy subjects. Chemotherapy induced an immediate fall in Hb (P less than 0.003), increase in S-iron (P less than 0.003) and transferrin saturation (P less than 0.001). Later in the disease a fall in S-transferrin (P less than 0.006) and an increase in S-ferritin (P less than 0.02) occurred. Thirty patients died during the 2 years observation. S-ferritin at discovery was correlated to performance status score (r = 0.57, P = 0.01) and to survival (r = -0.63, P less than 0.0002). Patients with S-ferritin less than or equal to 400 micrograms l-1 (n = 13) had longer survival than those with S-ferritin greater than 400 micrograms l-1 (n = 18) (P = 0.004).


Br. J. Cancer (1991), 64, 895-898 © Macmillan Press Ltd., 1991

### Iron status markers in patients with small cell carcinoma of the lung. Relation to survival

N. Milman[1,4], H. Sengeløv[2] & P. Dombernowsky[3]

[1]Department of Pulmonary Medicine, Gentofte Hospital, [2]Departments of Medicine C and Clinical Chemistry, Bispebjerg Hospital; [3]Department of Oncology, Herlev Hospital, Copenhagen; [4]Department of Clinical Chemistry, Bornholm Central Hospital, Rønne, Denmark.

**Summary** A longitudinal study of iron status markers (haemoglobin (Hb), serum (S-) iron, S-transferrin, transferrin saturation, S-ferritin) was performed in 31 chemotherapy treated patients with small cell lung cancer. At discovery, eight patients were anaemic (Hb $< 121$ g $l^{-1}$). Hb, S-iron and transferrin saturation were lower ($P < 0.01$), and S-ferritin higher ($P < 0.01$) than in healthy subjects. Chemotherapy induced an immediate fall in Hb ($P < 0.003$), increase in S-iron ($P < 0.003$) and transferrin saturation ($P < 0.001$). Later in the disease a fall in S-transferrin ($P < 0.006$) and an increase in S-ferritin ($P < 0.02$) occurred. Thirty patients died during the 2 years observation. S-ferritin at discovery was correlated to performance status score ($r = 0.57$, $P = 0.01$) and to survival ($r = -0.63$, $P < 0.0002$). Patients with S-ferritin $\leqslant 400\ \mu g\ l^{-1}$ ($n = 13$) had longer survival than those with S-ferritin $> 400\ \mu g\ l^{-1}$ ($n = 18$) ($P = 0.004$).

Patients with untreated malignant disease demonstrate characteristic changes in iron metabolism with anaemia, low serum (S-) iron and S-transferrin together with inappropriately elevated S-ferritin (Groop *et al.*, 1978; Lee, 1983). In general, the administration of cytotoxic agents induces a further fall in haemoglobin (Hb), and an increase in S-iron levels (Alfrey *et al.*, 1966; Doll *et al.*, 1983; Grau *et al.*, 1985; Pollera *et al.*, 1987). The pathophysiological mechanisms of these changes in iron kinetics are unclarified.

The aim of the present investigation was to study changes in iron status markers in patients with small cell carcinoma of the lung (SCCL), and to evaluate their relation to survival.

### Material and methods

The material comprised 34 consecutive patients with SCCL; two were excluded due to short survival < 1 month, and one due to porphyria cutanea tarda with iron overload. Median age in the included 31 patients (24 males, seven females) was 64 years (range 39-76). Prior to treatment, bone marrow and liver biopsies were performed to evaluate the stage of disease; 19 patients had limited disease, i.e. confined to one hemithorax including bilateral supraclavicular lymph nodes. Twelve patients had extensive disease, four of these had bone marrow involvement. Performance status score (PS) was assessed according to WHO criteria (WHO, 1979). All patients received combination chemotherapy consisting of lomustine, cyclophosphamide, methotrexate, vincristin, etoposide and doxorubicin in limited disease (Østerlind *et al.*, 1991), and of lomustine, cyclophosphamide, vincristin and etoposide in extensive disease.

None took iron supplementation. Blood transfusions were registered. Blood samples (non-fasting) were taken prior to chemotherapy, and every 4th week until death. Hb was measured on Coulter-S (1 g Hb $l^{-1} = 0.062$ mmol Hb $l^{-1}$), S-iron by spectrophotometry (Iron Test Roche[R] on Kobas Bio[R]), S-transferrin by immunoelectrophoresis, and S-ferritin by radioimmunoassay (Ferritin RIA Amersham[R]). Biochemical liver tests (S-aspartate aminotransferase and S-alkaline phosphatase) were measured by standard methods. The control material consisted of 103 healthy 65-year-old subjects (55 males, 48 females) recruited through a population study in Copenhagen County (Milman *et al.*, GEN-MONICA study, unpublished results). Iron status markers were measured by the same methods as in the present study.

In the statistical analysis, Wilcoxon's rank sum test for paired values was employed to evaluate the significance of differences, and correlations were assessed with Pearson's coefficient of correlation. Cumulative survival rates were calculated by the Kaplan-Meier method and compared statistically by the log rank test.

### Results

One male patient survived for more than 2 years. Median survival in the other 30 patients was 337 days (range 86-576). Neither Hb nor iron status indicators displayed any significant sex difference in the SCCL patients, and due to the small number of females, values in both sexes were pooled (Table I, Figures 1-2).

### *Haemoglobin*

Eight patients (26%) had Hb values $< 121$ g $l^{-1}$ (7.5 mmol $l^{-1}$) at discovery, *vs* 12 (40%) at the last measurement before death. There was a marked decline in Hb levels during the disease, the major fall occurring 1 month after initiation of chemotherapy (Table I, Figure 1).

### *Iron status markers*

*S-iron* showed a significant increase from discovery to death. The major increase was observed within 1 month after initiation of chemotherapy. *S-transferrin* demonstrated a decline occurring gradually and being significant from the third month of survival. *Transferrin saturation* showed a significant increase, being most pronounced in the first 3-4 months after initiation of chemotherapy. None of the patients displayed values $< 15\%$. High values $> 60\%$ were observed in 5 (17%) patients shortly before death. *S-ferritin* demonstrated a significant rise, which occurred late in the disease, after 10-11 months of survival (Figure 2). The distributions of S-ferritin values are shown in Table II. None of the patients had values $\leqslant 20\ \mu g\ l^{-1}$, and 16 (53%) had values $> 300\ \mu g\ l^{-1}$ at discovery compared to 29 (97%) at death.

### *Blood transfusions*

The long term survivor received 42 transfusions, while 22 patients had a median of four transfusions (range 2-12).

Correspondence: N. Milman, Department of Pulmonary Medicine Y, Gentofte Hospital, DK-2900 Hellerup, Denmark.
Received 28 January 1991; and in revised form 20 June 1991.

**Table I** Haemoglobin and iron status markers (median and 5–95 percentile) in patients with SCCL and in healthy subjects

| *Patients* | *Hb* $(g\,l^{-1})$ | *S-iron* $(\mu mol\,l^{-1})$ | *S-transferrin* $(\mu mol\,l^{-1})$ | *Transferrin saturation (%)* | *S-ferritin* $(\mu g\,l^{-1})$ |
|---|---|---|---|---|---|
| | 134[a] | 11[a] | 28 | 21[a] | 314[a] |
| Discovery | (110–153) | (4–20) | (19–35) | (11–32) | (51–1630) |
| ($n$ = 31) | <0.01 | <0.05 | <0.01 | <0.01 | <0.01 |
| *P* | 111[a] | 15[b] | 21[a] | 34 | 1128[a] |
| Death | (93–129) | (4–38) | (15–32) | (13–94) | (337–2124) |
| ($n$ = 30) | | | | | |
| Healthy subjects | ♂ 148 | 19 | 30 | 30 | ♂ 150 |
| (♂ = 55) | (124–171) | (11–30) | (21–36) | (17–50) | (62–725) |
| (♀ = 48) | ♀ 140 | | | | ♀ 107 |
| | (116–158) | | | | (42–257) |

Patients *vs* healthy subjects. [a]$P<0.01$; [b]$P<0.05$.

**Figure 1** Changes in serum iron, serum transferrin, transferrin saturation and haemoglobin during course of disease in 31 patients with SCCL (mean ± s.e.m.).

**Figure 2** Changes in serum ferritin levels during course of disease in 31 patients with SCCL (mean ± s.e.m.).

There was no correlation between the number of transfusions and iron status markers at death.

*Biochemical liver tests*

At discovery 16 (52%) patients had elevated S-transaminase and/or S-alkaline phosphatase. Iron status markers in patients with abnormal biochemical liver tests were not significantly different from those in patients with normal liver tests.

*Stage of disease*

There was no significant difference in iron status markers between patients with limited and extensive disease.

*Correlation between iron status markers*

At discovery, correlations existed between S-ferritin *vs* S-transferrin ($r = -0.48$, $P<0.01$), Hb *vs* S-iron ($r = 0.58$, $P<0.0005$), and Hb *vs* S-transferrin ($r = 0.42$, $P<0.02$).

*Survival*

PS was correlated to survival ($r = -0.37$, $P = 0.05$). S-transferrin at discovery was correlated to survival ($r = 0.44$, $P<0.02$), but closer analysis showed that this relation was of no clinical relevance. S-ferritin at discovery showed a negative correlation to survival (log *vs* log values, $r = -0.63$, $P<0.0002$, Figure 3). Analysis of survival rates revealed that a threshold ferritin value of $400\,\mu g\,l^{-1}$ yielded the best discrimination between the two survival groups (Figure 4). Furthermore S-ferritin at discovery was correlated to PS ($r = 0.57$, $P = 0.01$). Iron status markers at death showed no correlation to survival.

### Discussion

Iron status in patients with SCCL was profoundly influenced by disease and chemotherapy. Prior to treatment, Hb values were low and many patients were anaemic. Chemotherapy was immediately followed by a decrease in *Hb* levels due to inhibition of erythropoiesis (Alfrey *et al.*, 1966); the addi-

**Table II** Distribution of serum ferritin values in patients with SCCL at discovery and prior to death

| *S-ferritin* $(\mu g\,l^{-1})$ | *No. of patients* Discovery $n = 31$ | Death $n = 30$ |
|---|---|---|
| $\leqslant = 20$ | 0 | 0 |
| 21–40 | 2 | 0 |
| 41–300 | 12 | 1 |
| >300 | 17 | 29 |

**Figure 3** Relation between survival and serum ferritin at discovery in 31 patients with SCCL; regression line: $y = -1.24 \times + 5.56$; $r = -0.63$, $P < 0.0002$.

**Figure 4** Survival curves for patients with newly discovered SCCL having serum ferritin $>400\,\mu g\,l^{-1}$ (—, $n = 13$), and $\leqslant 400\,\mu g\,l^{-1}$ (—, $n = 18$) ($P = 0.004$).

tional fall prior to death was probably caused by metastatic marrow invasion.

Before chemotherapy, the patients were hyposideraemic, consistent with the fact that malignant disease produce an impaired release of iron from the storage compartments and a fall in *S-iron* (Lee, 1983; Lipschitz *et al.*, 1971). Chemotherapy induced a rise in S-iron levels, as described in other investigations (Grau *et al.*, 1985; Pollera *et al.*, 1987). The rise begins already 24–48 h after initiation of therapy (Pollera *et al.*, 1987). Throughout the remaining period of disease, S-iron remained elevated. The reasons for these changes in S-iron are unknown. Haemolysis can be excluded according to previous studies (Doll *et al.*, 1983; Pollera *et al.*, 1987), and increased iron absorption or decreased excretion are unlikely to give these rapid fluctuations. A decreased Hb synthesis *per se* induces a moderate rise in S-iron, as erythropoiesis modulates the release of iron from storage compartments (Alfrey *et al.*, 1966).

*S-transferrin* values at discovery were of the same order as in healthy subjects, but declined after 3–4 months of disease. Malignancies are known to reduce S-transferrin levels, probably due to decreased synthesis combined with increased catabolism (Hughes *et al.*, 1972; Lee, 1983).

The increase in *transferrin saturation* was a consequence of the rise in S-iron, combined with an almost constant S-transferrin level. None of the patients had values $<15\%$, indicating insufficient iron delivery to the erythron. Later in the disease, there was a rise in saturation due to declining S-transferrin and a further increase in S-iron.

In healthy subjects, the *S-ferritin* concentration reflects mobilisable iron stores, mainly located in the liver, spleen and bone marrow (Walters *et al.*, 1973); values $\leqslant 20\,\mu g\,l^{-1}$ are indicative of 'exhausted', $21-40\,\mu g\,l^{-1}$ of 'small', $41-300\,\mu g\,l^{-1}$ of 'normal' and $>300\,\mu g\,l^{-1}$ of 'increased' iron stores (Milman *et al.*, 1983).

To some extent S-ferritin behaves as an acute phase reactant, being inappropriately elevated in infectious, inflammatory and malignant diseases (Gropp *et al.*, 1978; Lee, 1983). S-ferritin was elevated at discovery and remained relatively constant until late in the disease, where a marked rise was observed at 10–11 months, probably reflecting an increased tumour mass. The present ferritin assay uses antibodies against basic spleen ferritin, and thus underestimates the presence of acidic ferritin often produced in malignant tumours (Hazard *et al.*, 1977).

Prognostic indicators of survival are of importance to the clinician in the decision for treatment. In a study comprising 778 patients with SCCL (Østerlind *et al.*, 1986), a number of clinical and biochemical variables (not including iron status markers) were tested against survival. PS at the time of diagnosis appeared to be the most weighty single factor. In the present study, PS also correlated to survival. Furthermore, there was a pronounced correlation between S-ferritin at discovery and survival, which was higher than the correlation between PS and survival. A threshold S-ferritin value of $400\,\mu g\,l^{-1}$ divided the patients in groups with highly significant differences in survival, i.e. high ferritin levels were related to a short survival. Cox *et al.* (1986) also found a relation between S-ferritin and survival in SCCL, using a discriminative value of $600\,\mu g\,l^{-1}$. Whether S-ferritin may be of clinical significance in the future evaluation of the prognosis in patients with SCCL should be investigated in more comprehensive studies.

The study was supported by The Danish Cancer Society (grant no. 86-033).

### References

ALFREY, C.P. Jr., LANE, M. & KARJALA, R.J. (1966). Modification of ferrokinetics in man by cancer chemotherapeutic agents. *Cancer*, **19**, 428.

COX, R., GYDE, O.H. & LEYLAND, M.J. (1986). Serum ferritin levels in small cell lung cancer. *Eur. J. Cancer Clin. Oncol.*, **22**, 831.

DOLL, D.C. & WEISS, R.B. (1983). Chemotherapeutic agents and the erythron. *Cancer Treatment Rev.*, **10**, 185.

GRAU, J.J., ESTAPE, J., DANIELS, M., GUZMAN, M.C. & SANTABARBARA, P. (1985). Cisplatin and plasma iron levels. *Ann. Intern. Med.*, **103**, 158.

GROPP, C., HAVEMANN, K. & LEHMANN, F.-G. (1978). Carcinoembryogenic antigen and ferritin in patients with lung cancer before and during therapy. *Cancer*, **42**, 2802.

HAZARD, J.T., YOKOTA, M., AROSIO, P. & DRYSDALE, J.W. (1977). Immunological differences in human isoferritins: implications of immunologic quantitation of serum ferritin. *Blood*, **49**, 139.

HUGHES, N.R. (1972). Serum transferrin and ceruloplasmin concentrations in patients with carcinoma, melanoma, sarcoma and cancers of haematopoietic tissues. *Austral. J. Exp. Biol. Med. Sci.*, **50**, 97.

LEE, G.R. (1983). Anaemia of chronic disease. *Sem. Hematol.*, **20**, 61.

LIPSCHITZ, D.A., SIMON, M.O., LYNCH, S.R., DUGARD, J., BOTHWELL, T.H. & CHARLTON, R.W. (1971). Some factors affecting the release of iron from reticuloendothelial cells. *Br. J. Haematol.*, **21**, 289.

MILMAN, N., PEDERSEN, N.S. & VISFELDT, J. (1983). Serum ferritin in healthy Danes: relation to bone marrow haemosiderin iron. *Dan. Med. Bull.*, **30**, 115.

ØSTERLIND, K. & ANDERSEN, P.K. (1986). Prognostic factors in small cell lung cancer: multivariate model based on 778 patients treated with chemotherapy with or without irradiation. *Cancer. Res.*, **46**, 4189.

ØSTERLIND, K., HANSEN, M., HIRSCH, F.R., DOMBERNOWSKY, P., SÖRENSON, S., PEDERSEN, A.G. & HANSEN, H.H. (1991). Combination chemotherapy of limited stage small cell lung cancer. A controlled trial on 221 patients comparing two alternating regimens. *Ann. Oncol.*, **2**, 41.

POLLERA, C.F., AMEGLIE, F., REINA, S., NARDI, M., ABBOLITE, M.R. & PARACINE, C. (1987). Changes in serum iron levels following very high-dose cisplatin. *Cancer Chemother. Pharmacol.*, **19**, 257.

WALTERS, G.O., MILLER, F.M. & WORWOOD, M. (1973). Serum ferritin concentration and iron stores in normal subjects. *J. Clin. Pathol.*, **26**, 770.

WORLD HEALTH ORGANIZATION (1979). Handbook for reporting results of cancer treatment. WHO: Geneva.